# Supplementary material for: Treatment-Related Adverse Events of Combination EGFR Tyrosine Kinase Inhibitor and Immune Checkpoint Inhibitor in EGFR-Mutant Advanced Non-Small Cell Lung Cancer: A Systematic Review and Meta-Analysis
Source: Cancers (Basel). 2022 Apr 26;14(9):2157. doi: 10.3390/cancers14092157 (PMC9102470; doi:10.3390/cancers14092157)
Supplement: Supplementary file 1 [file cancers-14-02157-s001.zip › Supplementary materials/Supplementary Tables&Figures.pdf]

Supplementary File

Treatment-related Adverse Events of Combination EGFR Tyrosine Kinase Inhibitor and Immune Checkpoint Inhibitor in EGFR-mutant Advanced Non-small Cell Lung Cancer: A Systematic Review and Meta-Analysis

Supplementary Figure S1. Any-grade overall trAEs. (A) Combination therapy with epidermal growth factor tyrosine kinase inhibitor and immune checkpoint inhibitor. (B) Osimertinib monotherapy.

Supplementary Figure S2. Grade ≥3 overall trAEs. (A) Combination therapy with epidermal growth factor tyrosine kinase inhibitor and immune checkpoint inhibitor. (B) Osimertinib monotherapy.

Supplementary Figure S3. Any-grade skin trAEs. (A) Combination therapy with epidermal growth factor receptor tyrosine kinase inhibitor and immune checkpoint inhibitor. (B) Osimertinib monotherapy.

Supplementary Figure S4. Any-grade gastrointestinal trAEs. (A) Combination therapy with epidermal growth factor receptor tyrosine kinase inhibitor and immune checkpoint inhibitor. (B) Osimertinib monotherapy.

Supplementary Figure S5. Any-grade interstitial lung diseases. (A) Combination therapy with epidermal growth factor receptor tyrosine kinase inhibitor and immune checkpoint inhibitor. (B) Osimertinib monotherapy.

Supplementary Figure S6. Grade ≥3 skin trAEs. (A) Combination therapy with epidermal growth factor receptor tyrosine kinase inhibitor and immune checkpoint inhibitor. (B) Osimertinib monotherapy.

Supplementary Figure S7. Grade ≥3 gastrointestinal trAEs. (A) Combination therapy with epidermal growth factor receptor tyrosine kinase inhibitor and immune checkpoint inhibitor. (B) Osimertinib monotherapy.

Supplementary Figure S8. Grade ≥3 interstitial lung disease. (A) Combination therapy with epidermal growth factor receptor tyrosine kinase inhibitor and immune checkpoint inhibitor. (B) Osimertinib monotherapy.

Supplementary Figure S9. Sensitivity analysis on any-grade interstitial lung disease. (A) Combination therapy with epidermal growth factor receptor tyrosine kinase inhibitor and immune checkpoint inhibitor. (B) Osimertinib monotherapy, among studies with sample size ≥ 40.

Supplementary Figure S10. Sensitivity analysis on grade ≥3 interstitial lung disease. (A) Combination therapy with epidermal growth factor receptor tyrosine kinase inhibitor and immune checkpoint inhibitor. (B) Osimertinib monotherapy, among studies with sample size ≥ 40.

Supplementary Table S1. Summary of osimertinib monotherapy studies.

Supplementary Table S2. Ongoing clinical trials of immune checkpoint inhibitors (ICI) in advanced, metastatic non-small cell lung cancer.

Supplementary Table S3. Incidence and nature of trAEs in selected clinical studies.

Supplementary Table S4. The Cochrane Collaboration's tool for assessing risks of bias of randomized-controlled trials.

Supplementary Table S5. Newcastle-Ottawa Scale (NOS) for quality assessment of non-randomized-controlled trials.

Supplementary Figure S1. Any-grade overall trAEs. (A) Combination therapy with epidermal growth factor tyrosine kinase inhibitor and immune checkpoint inhibitor. (B) Osimertinib monotherapy.

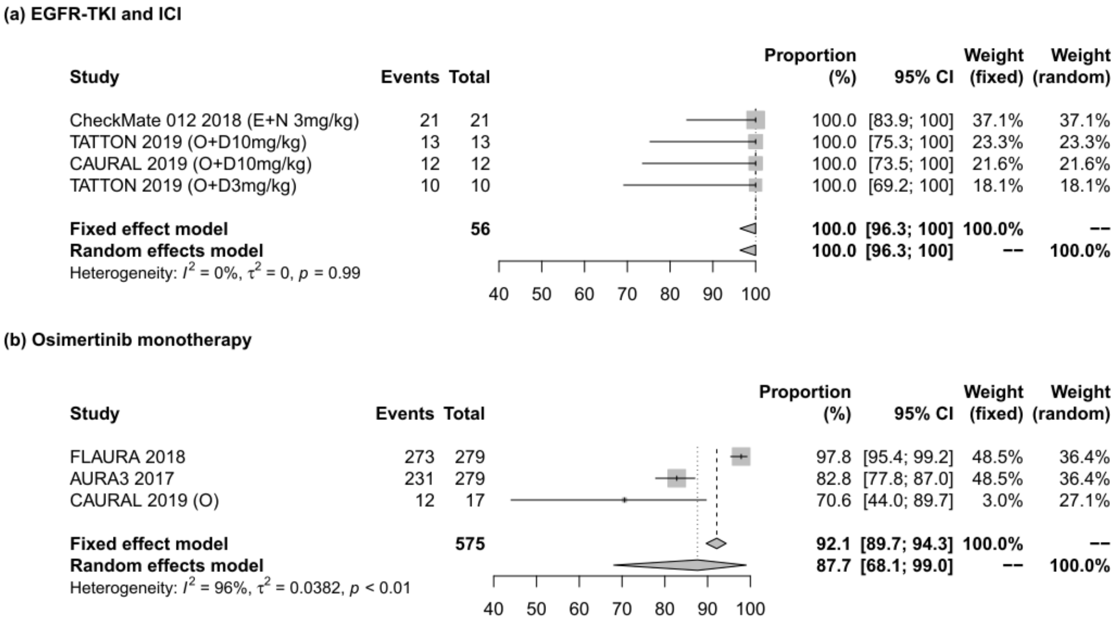

The squares represent the proportions of AE from each study, with smaller squares for larger sample size of the studies. The diamonds represent pooled proportions generated from fixed effect or random effects model. D, durvalumab; E, erlotinib; EGFR, epidermal growth factor receptor; ICI, immune checkpoint inhibitor; N, nivolumab; O, osimertinib; trAEs, treatment-related adverse events; TKI, tyrosine kinase inhibitor.

Supplementary Figure S2. Grade ≥3 overall trAEs. (A) Combination therapy with epidermal growth factor tyrosine kinase inhibitor and immune checkpoint inhibitor. (B) Osimertinib monotherapy.

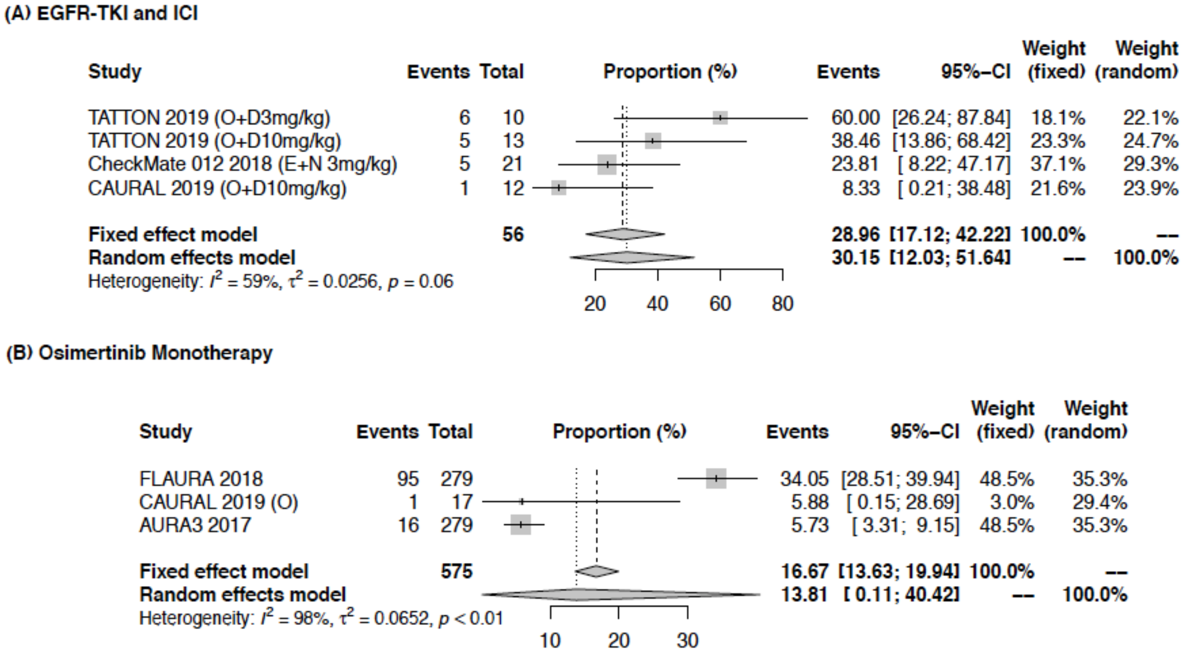

The squares represent the proportions of AE from each study, with smaller squares for larger sample size of the studies. The diamonds represent pooled proportions generated from fixed effect or random effects model. D, durvalumab; E, erlotinib; EGFR, epidermal growth factor receptor; ICI, immune checkpoint inhibitor; N, nivolumab; O, osimertinib; trAEs, treatment-related adverse events; TKI, tyrosine kinase inhibitor.

Supplementary Figure S3. Any-grade skin trAEs. (A) Combination therapy with epidermal growth factor receptor tyrosine kinase inhibitor and immune checkpoint inhibitor. (B) Osimertinib monotherapy.

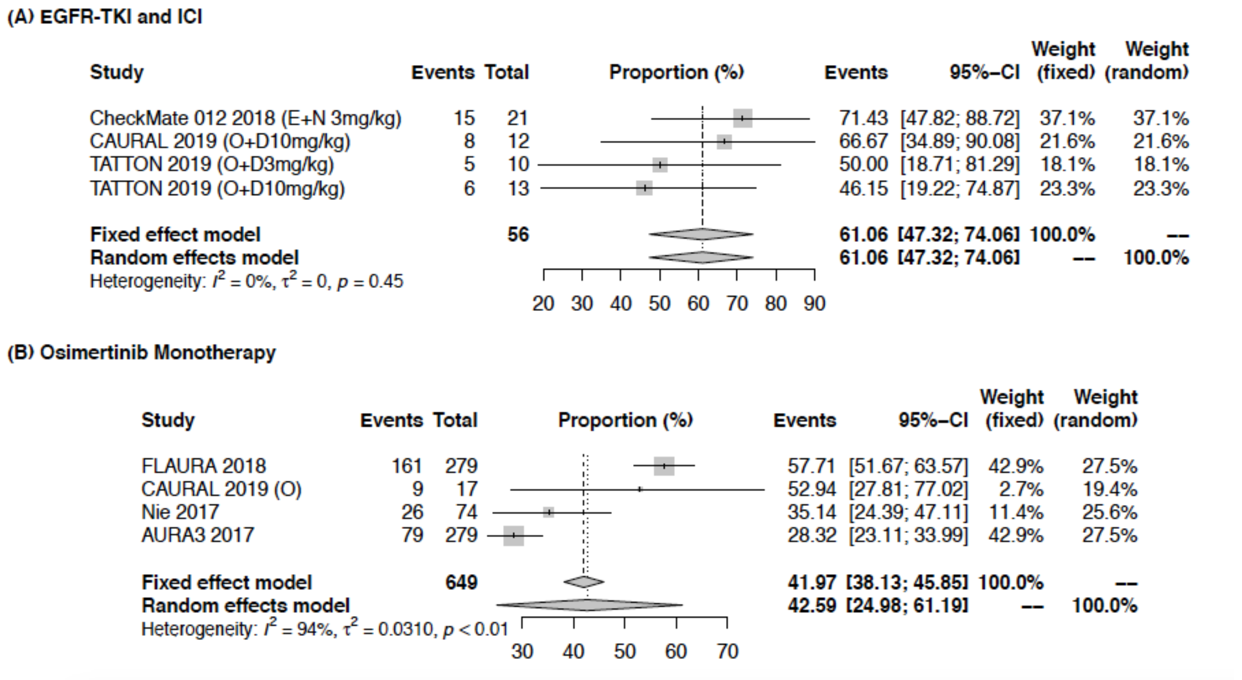

The squares represent the proportions of AE from each study, with smaller squares for larger sample size of the studies. The diamonds represent pooled proportions generated from fixed effect or random effects model. D, durvalumab; E, erlotinib; EGFR, epidermal growth factor receptor; ICI, immune checkpoint inhibitor; N, nivolumab; O, osimertinib; trAEs, treatment-related adverse events; TKI, tyrosine kinase inhibitor.

Supplementary Figure S4. Any-grade gastrointestinal trAEs. (A) Combination therapy with epidermal growth factor receptor tyrosine kinase inhibitor and immune checkpoint inhibitor. (B) Osimertinib monotherapy.

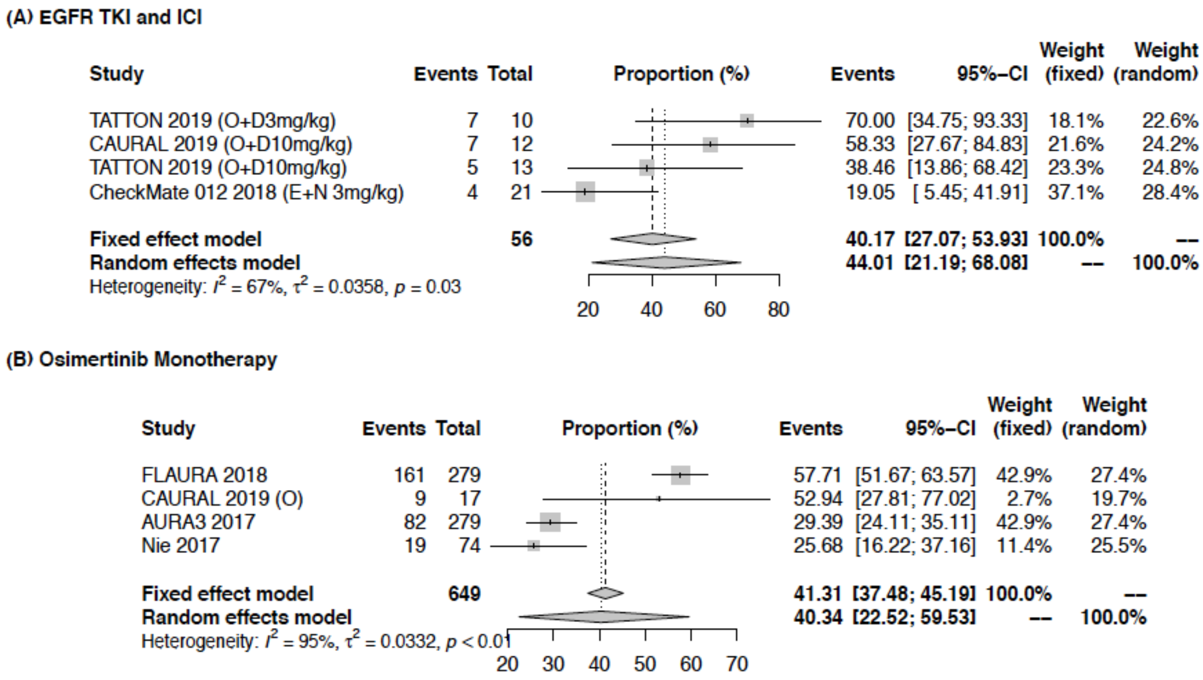

The squares represent the proportions of AE from each study, with smaller squares for larger sample size of the studies. The diamonds represent pooled proportions generated from fixed effect or random effects model. D, durvalumab; E, erlotinib; EGFR, epidermal growth factor receptor; ICI, immune checkpoint inhibitor; N, nivolumab; O, osimertinib; trAEs, treatment-related adverse events; TKI, tyrosine kinase inhibitor.

Supplementary Figure S5. Any-grade interstitial lung diseases. (A) Combination therapy with epidermal growth factor receptor tyrosine kinase inhibitor and immune checkpoint inhibitor. (B) Osimertinib monotherapy.

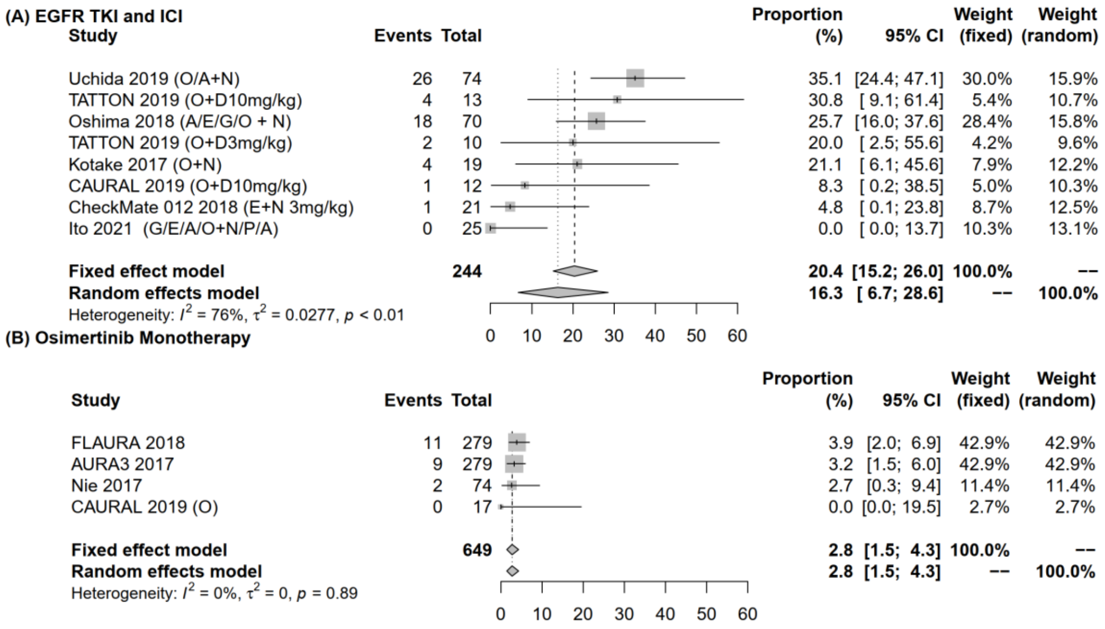

The squares represent the proportions of AE from each study, with smaller squares for larger sample size of the studies. The diamonds represent pooled proportions generated from fixed effect or random effects model. Af, afatinib; At, atezolizumab; D, durvalumab; E, erlotinib; EGFR, epidermal growth factor receptor; G, gefitinib; ICI, immune checkpoint inhibitor; N, nivolumab; O, osimertinib; P, pembrolizumab; trAEs, treatment-related adverse events; TKI, tyrosine kinase inhibitor.

Supplementary Figure S6. Grade ≥3 skin trAEs. (A) Combination therapy with epidermal growth factor receptor tyrosine kinase inhibitor and immune checkpoint inhibitor. (B) Osimertinib monotherapy.

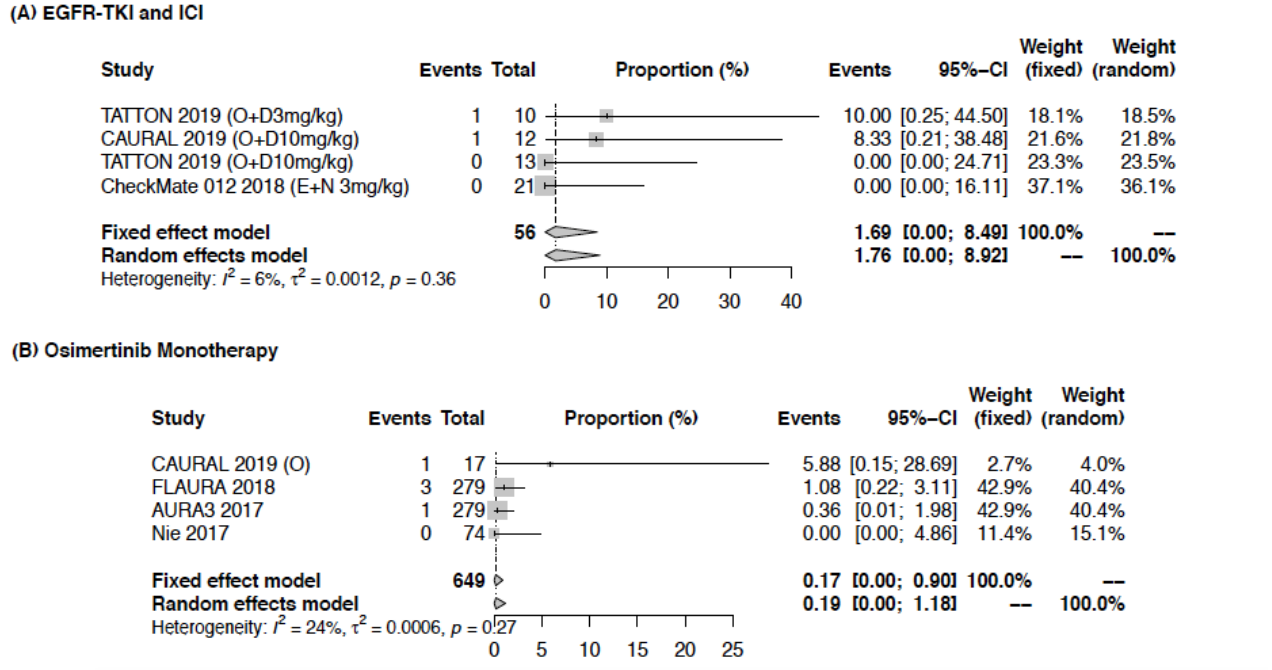

The squares represent the proportions of AE from each study, with smaller squares for larger sample size of the studies. The diamonds represent pooled proportions generated from fixed effect or random effects model. D, durvalumab; E, erlotinib; EGFR, epidermal growth factor receptor; ICI, immune checkpoint inhibitor; N, nivolumab; O, osimertinib; trAEs, treatment-related adverse events; TKI, tyrosine kinase inhibitor.

Supplementary Figure S7. Grade ≥3 gastrointestinal trAEs. (A) Combination therapy with epidermal growth factor receptor tyrosine kinase inhibitor and immune checkpoint inhibitor. (B) Osimertinib monotherapy.

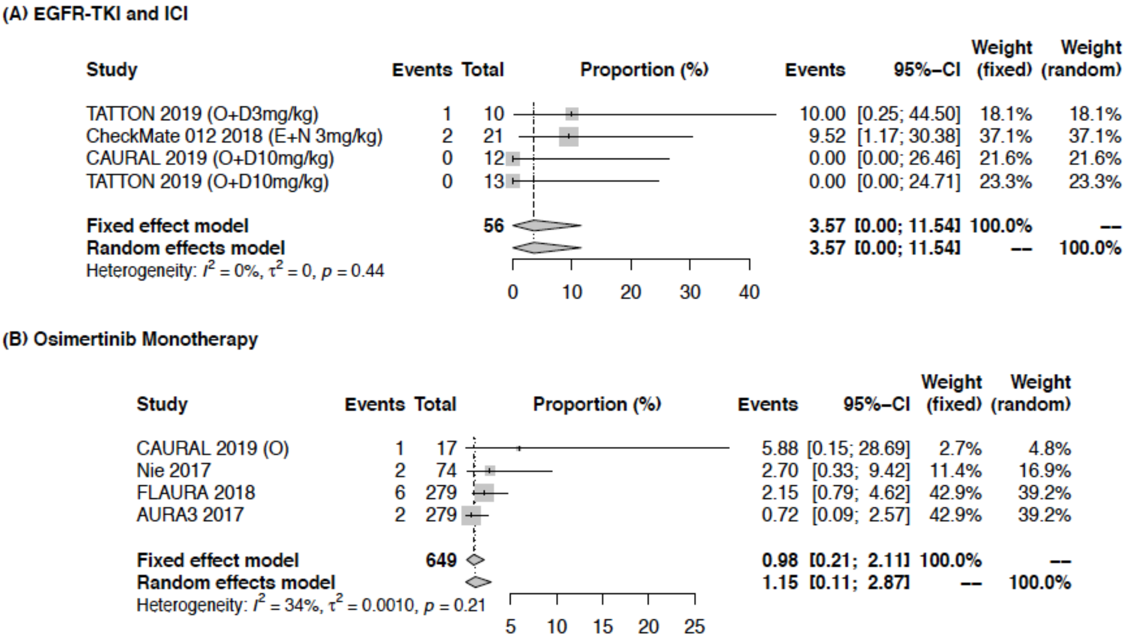

The squares represent the proportions of AE from each study, with smaller squares for larger sample size of the studies. The diamonds represent pooled proportions generated from fixed effect or random effects model. D, durvalumab; E, erlotinib; EGFR, epidermal growth factor receptor; ICI, immune checkpoint inhibitor; N, nivolumab; O, osimertinib; trAEs, treatment-related adverse events; TKI, tyrosine kinase inhibitor.

Supplementary Figure S8. Grade ≥3 interstitial lung disease. (A) Combination therapy with epidermal growth factor receptor tyrosine kinase inhibitor and immune checkpoint inhibitor. (B) Osimertinib monotherapy.

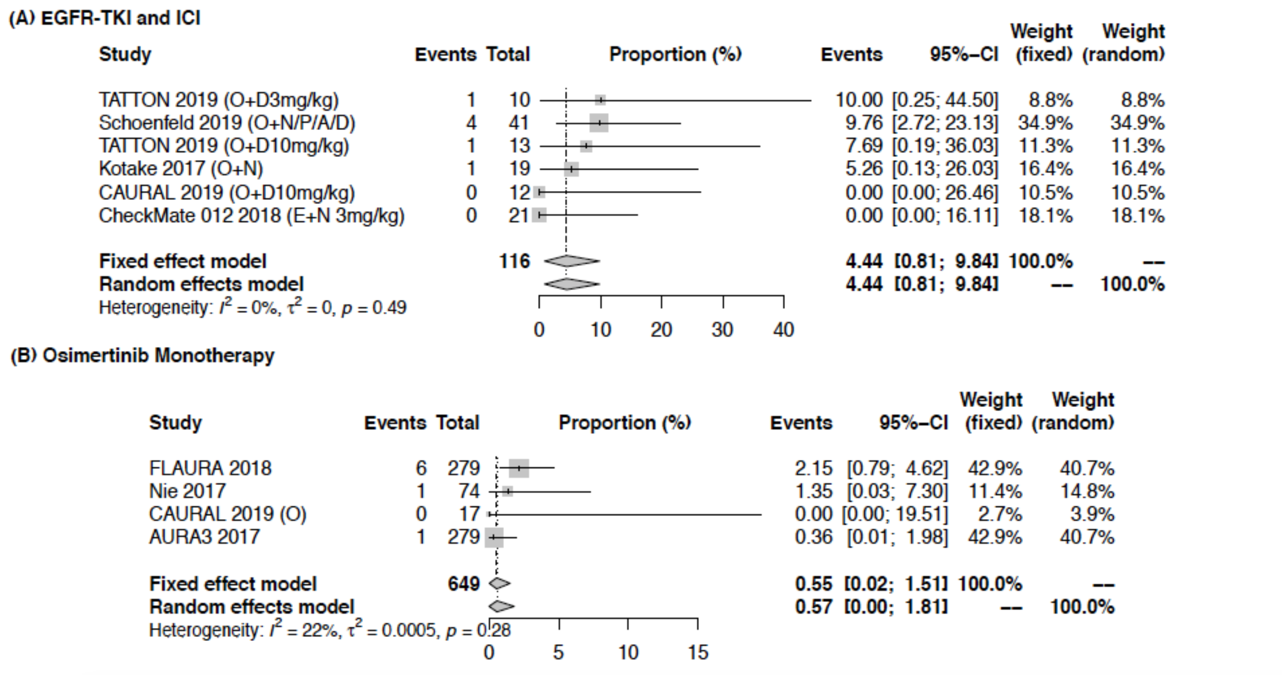

The squares represent the proportions of AE from each study, with smaller squares for larger sample size of the studies. The diamonds represent pooled proportions generated from fixed effect or random effects model. A, atezolizumab; D, durvalumab; E, erlotinib; EGFR, epidermal growth factor receptor; ICI, immune checkpoint inhibitor; N, nivolumab; O, osimertinib; P, pembrolizumab; trAEs, treatment-related adverse events; TKI, tyrosine kinase inhibitor.

Supplementary Figure S9. Sensitivity analysis on any-grade interstitial lung disease. (A) Combination therapy with epidermal growth factor receptor tyrosine kinase inhibitor and immune checkpoint inhibitor. (B) Osimertinib monotherapy, among studies with sample size ≥ 40.

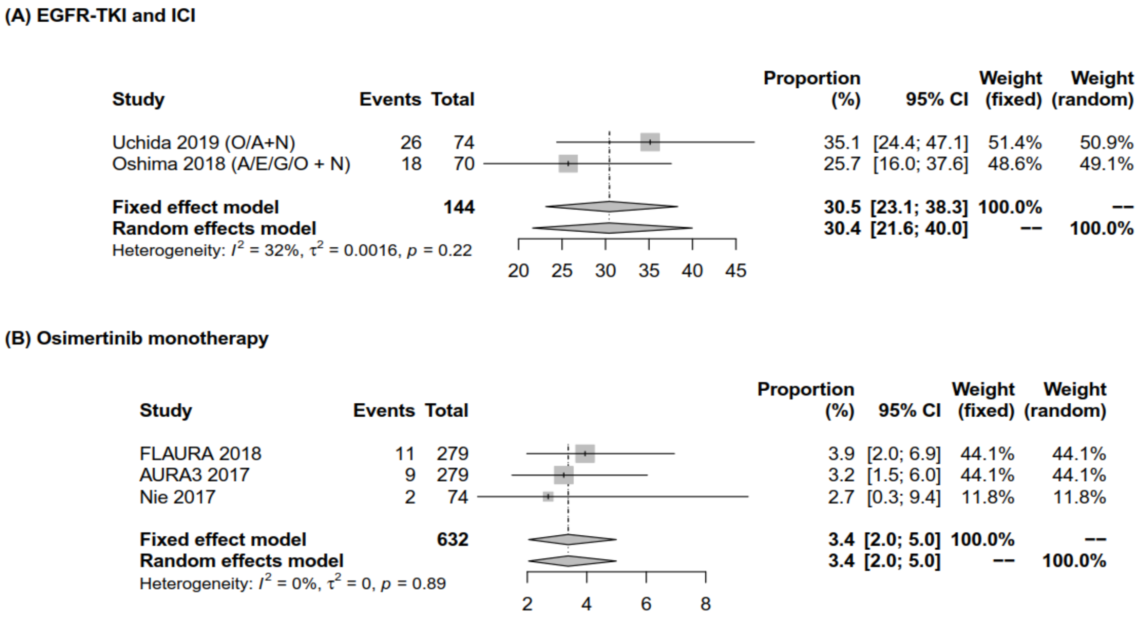

The squares represent the proportions of AE from each study, with smaller squares for larger sample size of the studies. The diamonds represent pooled proportions generated from fixed effect or random effects model. A, afatinib; D, durvalumab; E, erlotinib; EGFR, epidermal growth factor receptor; G, gefitinib; ICI, immune checkpoint inhibitor; N, nivolumab; O, osimertinib; trAEs, treatment-related adverse events; TKI, tyrosine kinase inhibitor.

Supplementary Figure S10. Sensitivity analysis on grade ≥3 interstitial lung disease. (A) Combination therapy with epidermal growth factor receptor tyrosine kinase inhibitor and immune checkpoint inhibitor. (B) Osimertinib monotherapy, among studies with sample size ≥ 40.

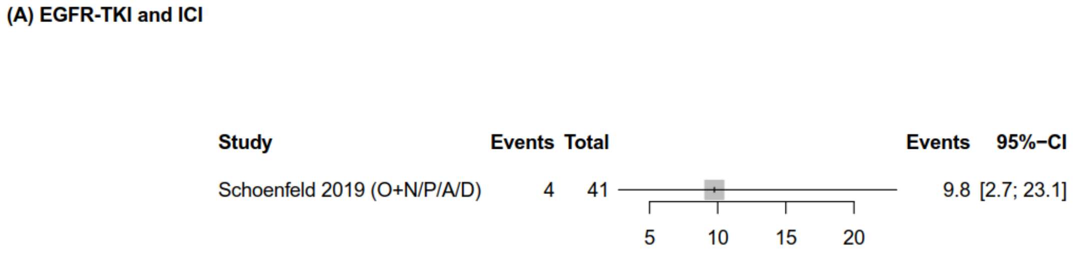

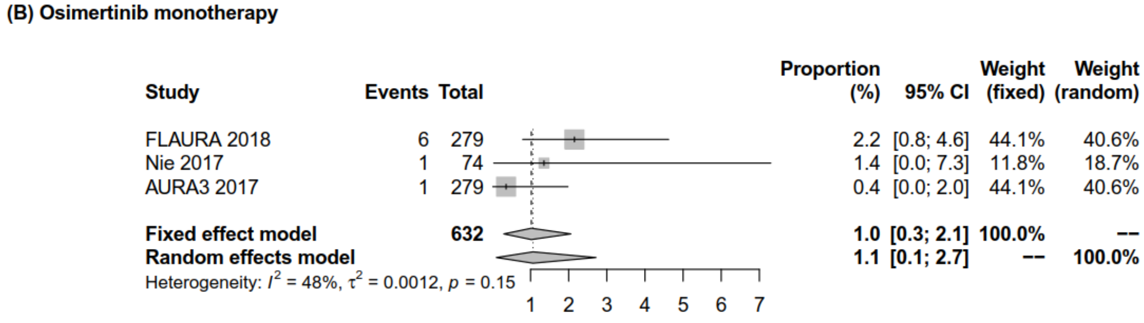

The squares represent the proportions of AE from each study, with smaller squares for larger sample size of the studies. The diamonds represent pooled proportions generated from fixed effect or random effects model. A, atezolizumab; D, durvalumab; EGFR, epidermal growth factor receptor; ICI, immune checkpoint inhibitor; O, osimertinib; P, pembrolizumab; trAEs, treatment-related adverse events; TKI, tyrosine kinase inhibitor.

Supplementary Table S1. Summary of osimertinib monotherapy studies.

Study	Year	Trial design	Subcategory	EGFR mutant (%)	Treatment line	Age (years)	Sample size (female %)	Dosage and length of osimertinib
Mok	2017	RCT Phase III	AURA3	T790M (100%)	Second	20–90	279 (62%)	80 mg daily until progression
Soria	2018	RCT Phase III	FLAURA	Ex19del/L858R (100%)1	First	26–93	279 (64%)	80 mg daily until progression
Marinis	2017	Single-arm Phase III b	ASTRIS	T790M (100%)	Second	27–92	1,217 (67%)	80 mg daily until progression
Nie	2017	RCT Phase III	NR	T790M (100%)	Third	18–80	74 (NR)	80 mg daily until progression
Planchard	2016	NR	NR	T790M (100%)	≥Second	28–92	350 (67%)	NR
Hochmair	2017	NR	NR	T790M (100%)	Second	NR	82 (NR)	80 mg daily until progression

NR, not reported.

Supplementary Table S2. Ongoing clinical trials of immune checkpoint inhibitors (ICI) in advanced, metastatic non-small cell lung cancer.

Clinical trial	Phase	Setting	Intervention	Status
NCT02364609	I	Chemotherapy or TKI-pretreated	Pembrolizumab + afatinib	Completed
NCT02013219	Ib	TKI naive	Atezolizumab + Erlotinib or Alectinib	Completed
NCT02179671	IIa	Exclude patients pretreated with ICI	Gefitinib, osimertinib, or selumetinib + docetaxel or tremelimumab with a sequential switch to durvalumab	Completed
NCT04245085/ ABC-lung	II	TKI-pretreated	Atezolizumab + bevacizumab + carboplatin-paclitaxel/ pemetrexed	Active, recruiting
NCT03647956	II	TKI-pretreated	Atezolizumab + bevacizumab + carboplatin +pemetrexed	Recruitment status unknown
NCT04042558	II	TKI-pretreated, ALK/ EGFR mutated or ROS1 fusion	Atezolizumab + carboplatin/ cisplatin + pemetrexed +/- bevacizumab	Recruiting
NCT04147351	II	TKI-pretreated, EGFR mutated	Atezolizumab + bevacizumab + carboplatin/ cisplatin + pemetrexed	Recruiting
NCT03513666	II	TKI-pretreated	Toripalimab + pemetrexed + carboplatin	Active, not recruiting
NCT03944772 ORCHARD	II	Osimertinib-pretreated, EGFR mutated	Durvalumab + carboplatin + pemetrexed (module 4), durvalumab + etoposide + carboplatin/ cisplatin (module 7)	Recruiting
NCT03994393/ ILLUMINATE	II	TKI-pretreated, EGFR mutated	Durvalumab and tremelimumab	Recruiting

EGFR, epidermal growth factor receptor; ICI, immune checkpoint inhibitor; TKI, tyrosine kinase inhibitor.

Supplementary Table S3. Incidence and nature of trAEs in selected clinical studies.

	Oxnard (TATTON)				Gettinger		Yang (CAURAL)			
	Osimertinib 80mg daily/ durvalumab 3mg/kg Q2W (n = 10)		Osimertinib 80mg daily/ durvalumab 10mg/kg Q2W (n = 13)		Nivolumab 3 mg/kg Q2W and erlotinib 150 mg daily (n = 21)		Osimertinib (80 mg once daily) (n = 17)		Osimertinib (80 mg once daily) with durvalumab (10 mg/kg IV Q2W) (n = 12)	
Treatment related adverse events	Any grade	Grade 3/4	Any grade	Grade 3/4	Any grade	Grade 3/4	Any grade	Grade 3/4	Any grade	Grade 3/4
Total (any grade AE)	21%	NA	100%	NA	100%	NA	100%	NA	100%	NA
Total (grade 3/4 AE)	NA	60%	NA	38.5%	NA	24%	NA	53%	NA	8%
Skin toxicity										
Dry skin (grouped terms)	30%	0	30.8%	0	19%	0	18%	0	33%	8%
Pruritis	NR	NR	NR	NR	19%	0	24%	0	42%	0
Rash (grouped terms)	50%	10%	46%	0	48%	0	53%	0	67%	0
Stomatitis	NR	NR	NR	NR	NR	NR	29%	0	0	0
Paronychia (grouped terms)	NR	NR	NR	NR	29%	0	41%	0	17%	0
Skin fissures	NR	NR	NR	NR	24%	0	NR	NR	NR	NR
Alopecia	NR	NR	NR	NR	14%	NR	NR	NR	NR	NR
Nail disorder	NR	NR	NR	NR	14%	NR	NR	NR	NR	NR
Hepatotoxicity										
ALT increased	NR	NR	NR	NR	14%	5%	NR	NR	NR	NR
AST increased	NR	NR	NR	NR	14%	10%	12%	0	25%	0
ALT/AST increased	NR	NR	NR	NR	NR	NR	NR	NR	NR	NR
Gastrointestinal toxicity										
Diarrhoea	30%	0	23%	0	19%	10%	53%	6%	60%	0
Nausea	30%	0	38%	0	19%	0	18%	0	8%	0
Vomiting	70%	10%	15%	0	14%	0	NR	NR	NR	NR
Decreased appetite	30%	10%	31%	0	0	0	12%	0	50%	0

Dry mouth	NR	NR	NR	NR	14%	0	1%	0	0	0
Colitis	NR	NR	NR	NR	0	0	NR	NR	NR	NR
Constipation	30%	0	23.1%	0	0	0	18%	0	33%	0
Pulmonary toxicity										
ILD/pneumonitis	20%	10%	31%	7.60%	5%	0	0	0	8%	0
Pneumonia	NR	NR	NR	NR	NR	NR	18%	12%	17%	0
Upper respiratory toxicity	0	0	0	0	NR	NR	24%	0	33%	
Cough	NR	NR	NR	NR	NR	NR	24%	0	33%	0
Upper respiratory infection	NR	NR	NR	NR	NR	NR	18%	0	8%	0
Viral upper respiratory infection	NR	NR	NR	NR	NR	NR	24%	0	8%	0
Dyspnea	NR	NR	NR	NR	NR	NR	24%	0	0	0
Productive cough	NR	NR	NR	NR	NR	NR	18%	0	8%	0
Rhinorrhea	NR	NR	NR	NR	NR	NR	18%	0	25%	0
General disorders and administration site conditions										
Pyrexia	20%	0	23%	7.70%	NR	NR	NR	NR	NR	NR
Fatigue	30%	0	23.1%	0	29%	0	NR	NR	NR	NR
Hypersensitivity (infusion) reaction	NR	NR	NR	NR	10%	0	NR	NR	NR	NR
Hematological toxicity										
Anemia	40%	0	31%	7.60%	NR	NR	NR	NR	NR	NR
Neutropenia	NR	NR	NR	NR	NR	NR	24%	12%	0	0
Neutrophil count decreased	NR	NR	NR	NR	NR	NR	18%	0	0	0
Endocrinopathies										
Hypothyroidism	NR	NR	NR	NR	14%	0	NR	NR	NR	NR
Acute thyroiditis	NR	NR	NR	NR	15%	0	NR	NR	NR	NR
Autoimmune thyroiditis	NR	NR	NR	NR	5%	0	NR	NR	NR	NR
Hyperthyroidism	NR	NR	NR	NR	5%	0	NR	NR	NR	NR
Rheumatological toxicity										
Arthralgia	NR	NR	NR	NR	NR	NR	29%	0	17%	0

Musculoskeletal toxicity										
Back pain	NR	NR	NR	NR	NR	NR	18%	0	25%	0
Neurological toxicity										
Hypoesthesia	NR	NR	NR	NR	NR	NR	6%	0	25%	0

NA; not applicable; NR, not reported; Q2W, every 2 weeks; trAEs, treatment-related adverse events.

Supplementary Table S4. The Cochrane Collaboration's tool for assessing risks of bias of randomized-controlled trials.

	Random sequence generation	Allocation concealment	Blinding of participants and personnel	Blinding of outcome assessment	Incomplete outcome data	Selective outcome reporting	Other sources of bias
CAURAL (2019)	+	?	-	-	+	+	+

The light green cells (+) indicate a low risk of bias. The red cells (-) indicate a high risk of bias. The yellow cells (?) indicate an uncertain risk of bias.

Supplementary Table S5. Newcastle-Ottawa Scale (NOS) for quality assessment of non-randomized-controlled trials.

	Selection	Comparability	Exposure/outcome	Overall star rating
TATTON (2019)	***	0	0	3
Gettinger (2018)	**	0	0	2

A star (*) system was used for allow a semi quantitative assessment of study quality. The NOS ranges from zero to nine stars. We considered high-quality studies as those that achieved seven or more stars, medium-quality studies those with four to six stars, and poor-quality studies those with only one to three stars.
